# *Ex vivo* investigation on internal tunnel approach/internal resin infiltration and external nanosilver-modified resin infiltration of proximal caries exceeding into dentin

**DOI:** 10.1371/journal.pone.0228249

**Published:** 2020-01-28

**Authors:** Andrej M. Kielbassa, Marlene R. Leimer, Jens Hartmann, Stephan Harm, Markus Pasztorek, Ina B. Ulrich

**Affiliations:** 1 Centre for Operative Dentistry, Periodontology, and Endodontology, University of Dental Medicine and Oral Health, Danube Private University, Krems, Austria; 2 Department for Biomedical Research, Danube University, Krems, Austria; Institute of Materials Science, GERMANY

## Abstract

This *ex vivo* proof-of-concept study aimed to investigate the effect of nanosilver particles (AgNP) added to a conventional infiltrant resin (Icon) on external penetration into natural proximal enamel caries exceeding into dentin after internal tunnel preparation and internal infiltration. Carious lesions (ICDAS codes 2/3) of extracted human (pre-)molars revealing proximal caries radiographically exceeding into dentin (E2/D1 lesions) were preselected. Then, 48 of those specimens showing demineralized areas transcending the enamel-dentin border as assessed by means of near-infrared light transillumination (DIAGNOcam) were deproteinized (NaOCl, 5%). Using an internal tunnel approach, occlusal cavities central to the marginal ridge were prepared. Excavation of carious dentin, total etch procedure (H_3_PO_4_, 40%), and internal resin infiltration (FITC-labeled) followed, along with final restorations (flowable composite resin). Outer lesion surfaces were etched (HCl, 15%) prior to external infiltration (RITC-labeled). Group 1 (control; n = 24) used non-modified infiltrant, while an infiltrant/AgNP mixture (20 nm; 5.5 wt%) was used with experimental Group 2 (n = 24). Non-infiltrated pores of cut lesions were stained (Berberine), and specimens were analyzed using confocal laser scanning microscopy. Compared to the non-filled infiltrant, incorporation of AgNP had no effect on the resin’s external penetration. Between the groups, no significant differences regarding internal or external infiltration could be detected, and non-infiltrated lesion areas did not differ significantly (p>0.109; *t*-test). The internal tunnel preparation in combination with both an internal resin infiltration and an additional external infiltration approach using a nanosilver-modified infiltrant resin leads to increased infiltrated lesion areas, thus occluding and adhesively stabilizing the porous volume of the demineralized enamel. While exerting antimicrobial effects by the nanosilver particles, this approach should have the potential as a viable treatment alternative for proximal lesions extending into dentin, thus avoiding the sacrifice of sound enamel, postponing the frequently inevitable restoration/re-restoration cycle of conventional proximal caries treatment, and improving dental health.

## Introduction

Minimal intervention is an indispensable element of modern dentistry focusing on preventive or non-surgical actions to preserve dental hard substances, thus avoiding any unnecessary sacrifice of tooth tissues, and ensuring a longest possible tooth survival [1]. Introduced in 2009, the resin infiltration technique using a low-viscosity resin originally has been developed with the intention to penetrate the demineralized and porous inter-crystalline spaces of initial subsurface enamel lesions, thereby occluding the latter after polymerization [2]. This ultraconservative approach effectively builds a covalently bound three-dimensional polymer framework [3, 4], thus (partially) replacing the lost minerals, encapsulating the hydroxyapatite crystals, micromechanically interlocking the remaining enamel prisms, and acting as an effective barrier for hydrogen ions to inhibit further demineralization and to arrest proximal subsurface lesion progress [4, 5].

Accordingly, timely systematic reviews on the clinical efficacy of this micro-invasive solution have confirmed its efficacy [6, 7]. With observation periods of up to four years in a clinical trial [8], the resin infiltration of proximal enamel lesions would seem to complement (or even outperform) other interventions like fluoridation and improved interdental hygiene [6, 9], even with high-risk caries patients [10, 11]. In fact, this approach does prevent from overtreatment, thus underpinning the suggested concept of a drill-less approach [12]. Consequently, a recently published guideline summarizing the respective literature has concluded that the resin infiltration concept is clinically feasible and reliable, and offers high success rates with non-cavitated proximal caries lesions restricted to enamel [13]. By implementing this technique, dental health will be maintained, and the use of surgical intervention will be reduced to a minimum, thus following the recommendations adopted by the International Caries Classification and Management System (ICCMS^**™**^) [14] and by CariesCare International (CCI^**™**^) [15].

Notwithstanding, initial enamel lesions are characterized by a reduced mineral content, thus leading to a decreased microhardness [16], and this in turn will result in a decreased stability, possibly jeopardizing the integrity of the respective regions; resin infiltration, however, has been reported to increase surface microhardness of demineralized human [17–19] or bovine [20–22] enamel significantly. All in all, this would suggest a recovered surface resistance of the respective areas [23]; nevertheless, it should be kept in mind that in particular with progressed enamel carious lesions the infiltration frequently will be inhomogeneous [24] and incomplete [3, 24] with respect to the total lesion depth. Not astonishingly, one of the recently published studies focusing on this topic could not reveal a reestablished microhardness considered comparable to sound enamel [25]. Thus, the infiltrated surface has not been shown to completely resist new cariogenic challenges [25, 26], and a previous review has emphasized that for caries extending into dentin, treatment efficacy of resin infiltration was not significantly different from the non-infiltrated controls [7]. Additionally (and not unexpectedly), a recently published randomized clinical trial has confirmed that the resin infiltrant’s capacity to arrest caries progression of lesions reaching the outer dentin is reduced to 64% [10], thus suggesting an only poor efficacy of resin infiltration for these advanced lesion types.

Obviously, surface microhardness is an inadequate single parameter to conclusively assess infiltrated caries lesions, and cross-sectional microhardness evaluations might be more meaningful [27]. Indeed, continuously decreasing cross-sectional hardness values of demineralized and infiltrated enamel have previously been shown with increasing lesion depths [23], and this would render non-infiltrated enamel lesion areas vulnerable. Moreover, it has recently been shown that the level of demineralization correlates with the presence of superficial microcracks within the vicinities of proximal contact areas [28]; hence, it does not seem surprising that the prevalence of marginal ridge fractures is associated with the presence of proximal carious lesions [29]. To overcome this fragility, a more comprehensive attempt to adhesively reinforce the enamel lesion would seem favorable [30, 31], and, therefore, a combined external/internal infiltration concept increasing the amount of infiltrated lesion volume has recently been introduced [32]. This treatment approach should further strengthen lesion resistance to (micro-)fractures.

Reflecting on proximal caries lesions extending (radiographically) into dentin and intended to be treated should raise a further consideration, namely that of the macroscopically non-cavitated (but nevertheless pre-damaged) surface of proximal caries. Indeed, previous papers have clarified that both the tendency of surface breakdown [33, 34] and the ICDAS codes [35, 36] will increase with advanced radiographic lesion extensions. Due to the material’s inherent mechanical properties [37], the infiltration approach using an unfilled and low-viscosity resin will not be able to completely fill up any (micro-)cavitations [2, 3, 38], nor will it be able to adequately smoothen the infiltrated surface of demineralized (and rough) enamel [39]; thus, several investigations have clearly elucidated that roughness of infiltrated lesions will remain increased if compared to sound enamel [18, 20, 21, 25, 40], and this will not be perfectible by various polishing procedures [41]. Rough surfaces (with R_a_ values exceeding 0.2 μm as the critical threshold), however, are susceptible to facilitate biofilm accumulation, and this has been revealed for infiltrated surfaces as well, even if to a lesser extent if compared to non-infiltrated lesions [17, 19, 42].

Therefore, adding filler particles exerting antibacterial properties to the low-viscosity resin might be a promising enhancement of the infiltration approach, and this should be interesting in particular for deeper lesions (reaching radiographically beyond the enamel-dentin junction), thus accidentally or intentionally surpassing the originally recommended indications for resin infiltration of non-cavitated enamel caries scored as ICDAS 1 and 2 (International Caries Detection and Assessment System) [35]. One possible prospective filler candidate would seem silver nanoparticles (AgNP) [43], ranging from 1 to 100 nm in diameter; in recent years, AgNP have been increasingly used for a wide range of applications in (nano-)medicine, and successful dental implementations have been reported as well [44, 45], including composite resins [46, 47]. The desired antimicrobial effects have been revealed [48, 49], and especially the long-lasting [50] and the long-distance bactericidal capability [51] should qualify AgNP as complimentary additives to infiltrant resins, thus preventing the latter from microbial re-colonization.

However, the available literature does not provide any information on possible effects of incorporated AgNP on the penetration ability of a resin infiltrant (Icon Caries Infiltrant; DMG, Hamburg, Germany). Thus, our objective with the present *ex vivo* investigation was to modify the resin infiltrant by using AgNP, and we hypothesized (H_0_) that the addition of AgNP would have no influence on the resin’s external penetration and the overall (internal/external) infiltration ability into non- and micro-cavitated proximal caries lesions (exceeding the enamel-dentin junction) of human premolars and molars. This null hypothesis was tested against the alternative hypothesis (H_1_) of a difference.

## Materials and methods

### Visual and radiographic selection of teeth

Extracted teeth were obtained from a company responsible for disposal of dental materials (Enretec, Velten, Germany), and human premolars and permanent molars showing chalky white/brownish incipient carious lesions on at least one proximal tooth surface were selected for the present study. Teeth revealing occlusal and/or proximal restorations as well as those showing visible fractures were excluded. In accordance with the German regulations of the Central Ethical Committee regarding the use of human body material in medicine [52], no ethical approval was mandatory, and we had unrestricted permission for the use of these anonymous teeth in research and for publication.

Prior to further use, all specimens were carefully cleaned from soft tissues and calculus (and with special care to avoid touching the carious lesion) using a dental ultrasonic device (Teneo; Dentsply Sirona, Bensheim, Germany). All teeth were preselected (MRL), independently classified visually according to the International Caries Detection and Assessment System (ICDAS) [35], and consented by three experienced observers (AMK, IBU, MRL) under ideal lighting conditions (Sirona C8+; Dentsply Sirona) with the naked eye; careful and pressureless probing [53] using a dental explorer (EXD3CH6; Hu-Friedy, Chicago, IL, USA) was used to exclude teeth obviously revealing frank or deep cavitations. For the current investigation, only proximal surfaces revealing ICDAS codes 2 and 3 were selected, and all teeth were dried with paper towels. Subsequently, the proximal caries lesions were photographed (E-M5 Mark II; lens 60 mm, 1:2.8, ringflash STF-8; Olympus, Hamburg, Germany).

**Fig 1:**
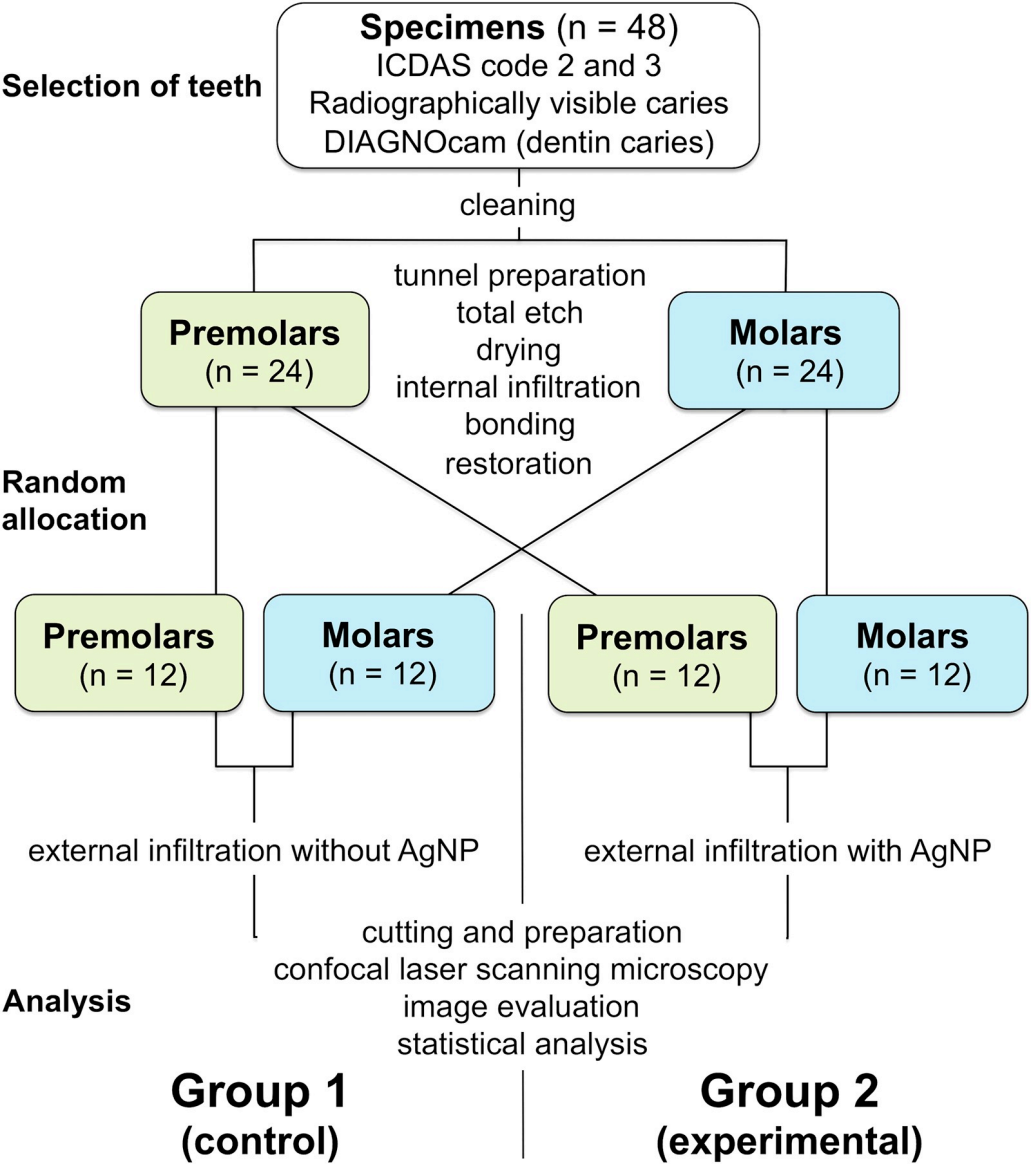
Flowchart presenting group assignment and experimental set-up.

To complement the (simulated clinical) evaluation, all selected teeth (initially consisting of 182 premolars and 122 molars) were radiographed by means of a commonly used X-ray system (0.08 s for premolars/0.10 s for molars; 60 kV; 7 mA; Heliodent plus; Dentsply Sirona) [32, 39]. To guarantee a reproduction of the respective positions, a wooden holder (in-house production) was used in combination with a surface bed made out of silicone (Silaplast; Detax, Ettlingen, Germany), to fix the X-ray tube. All teeth were perpendicularly attached in a flexible silicone base (Silaplast; Detax), with a 4-cm distance between their buccal aspects and the X-ray tube [33]. To simulate soft tissues, three Perspex panels (with a total dimension of 15 mm; Perspex Distribution, Chelmsford, UK) were fixed between tube and tooth as described previously [33, 39]. Subsequently, the radiological lesion depths were examined and consented by three examiners (AMK, IBU, MRL) using a six-point classification system [54]; only teeth revealing either E2 (translucency in the inner half of enamel) or D1 lesions (translucency in the outer third of dentin) were chosen. A flowchart presenting the study set-up is given with Fig 1.

### Confirmation of dentin caries using near-infrared transillumination

In addition, all teeth were screened by using a digital imaging near-infrared light transillumination (NILT) device for caries detection (DIAGNOcam; KaVo, Vienna, Austria), to double check the radiographic outcome as well as to determine the extension of lesion depths, viewed from the intact occlusal surface [55]. The teeth were placed in a gingival mask (elastic replacement gingiva, AN-4 WUKV; Frasaco, Tettnang, Germany), thus imitating clinical conditions. To fade out near-infrared radiation and ambient light, black colored (Edding 3000, 3 mm black; Edding, Ahrensburg, Germany) artificial teeth (n = 2; Frasaco) were fixed both mesially and distally using a glue gun (PSM Bestpoint, Wels, Austria). Before using the DIAGNOcam (KaVo), all teeth were immersed into saline solution (in-house production) for mimicking of natural saliva. After this confirmatory examination, only teeth showing an indisputable translucency in the outer third of dentin (comparable to radiographically visible D1 lesions) as diagnosed by means of the NILT device were chosen [56], and a total of 48 teeth (24 premolars, 24 molars) were selected. Then, all teeth were randomly divided into 2 groups, each comprising the same number of molars and premolars as well as the same number of ICDAS code 2 (n = 12) and code 3 (n = 12) lesions (Fig 1). Until further usage, the teeth were stored in 0.9% sodium chloride solution (0.9% NaCl solution; in-house production) at room temperature.

### Preparatory steps

**Fig 2:**
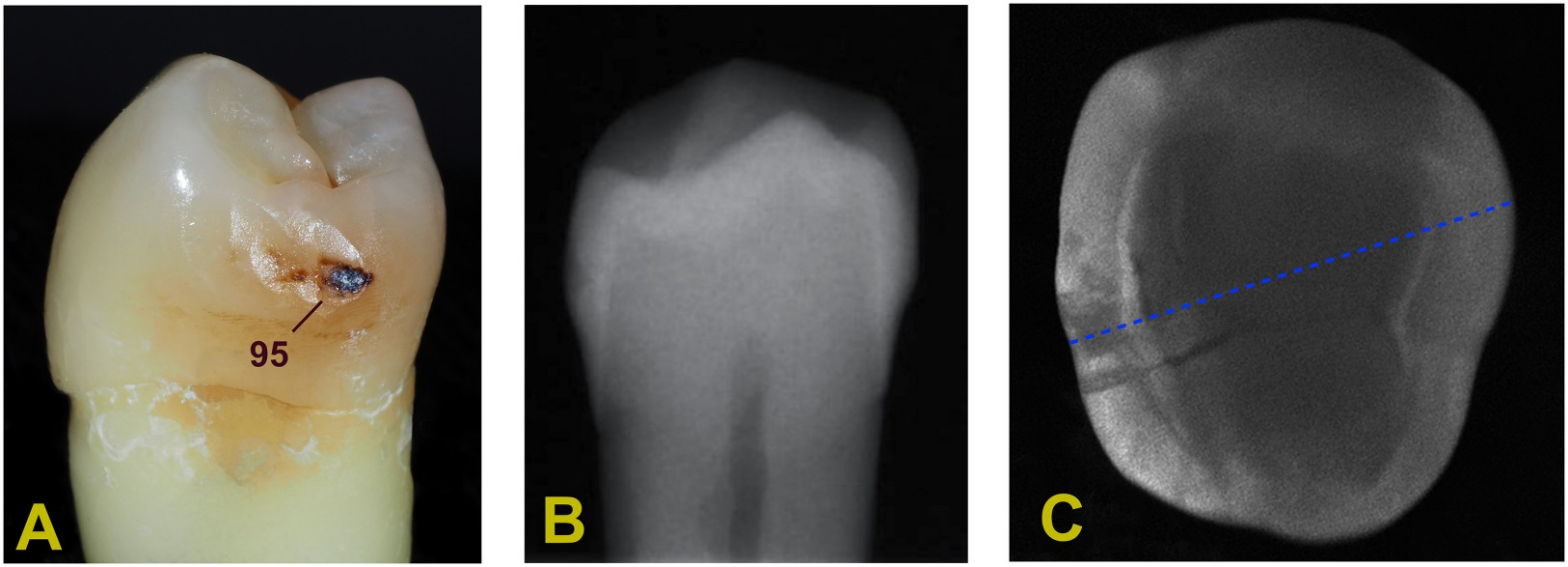
Representative example of premolar revealing proximal caries. Representative specimen of the experimental Group 2 (internal tunnel preparation as well as internal infiltration, and external infiltration using an infiltrant/nanosilver particle mixture). (**A**) Macroscopic view of incipient proximal caries lesion before treatment (DIAGNOdent pen value measured as indicated). (**B**) Radiograph of the respective specimen, revealing the proximal caries lesion not clearly extending into dentin. (**C**) Corresponding radiation-free DIAGNOcam image of the same specimen, depicting the extent of the carious lesion, along with the sectional plane for CLSM evaluation (indicated by the blue dotted line).

Subsequently, for all teeth the highest proximal demineralization areas of each tooth were defined with a calibrated laser fluorescence device (DIAGNOdent pen; KaVo) [32]. These areas were highlighted on printed photos of each lesion, and the respective marking points served as orientation for the cutting procedures described below. Finally, all chosen teeth were numbered and stored in hermetically sealed boxes (TO 706–12; Sogenex, Tood, Malo, Italy), filled with saline (in-house production) at room temperature. Fig 2 depicts a representative example of the clinical appearance (Fig 2A) of a specimen’s proximal caries, along with the respective radiographic (Fig 2B) and NILT views (Fig 2C).

After the finalization of the described selection process, all teeth were put into a plastic cup (Drinking Cups, #900–8366; Henry Schein, Melville, NY, USA), which was previously filled up with sodium hypochlorite (5%, NaOCl solution; Apotheke zum goldenen Engel, Graz, Austria) for deproteinization (20 min) of the outer surfaces. Then, all teeth (n = 48) were thoroughly cleaned with water spray using a multifunctional syringe (Sprayvit, Teneo; Dentsply Sirona) for 30 s, to remove any residuals of hypochlorite and dissolved organic material.

### Occlusal access preparation and internal infiltration

Using a red contra angle handpiece (160,000 rpm, C200 L, 1:5; Dentsply Sirona) in combination with permanent water cooling, occlusal cavities central to the marginal ridge were prepared using a minimally invasive diamond-coated bur (#830.314.010; Komet Austria, Salzburg, Austria) until the proximal caries of all teeth became apparent from the internal side. Then, after removing the dentin layers, the inner portion of the proximal lesion was uncovered from the central cavity. To gain access to the diseased enamel, a rose head bur (#H1SE.204.016; Komet Austria) was used without water cooling, driven by a green handpiece (10,00 rpm, S-Max, M15L, 4:1; Dentsply Sirona). All procedures were carried out using magnifying glasses (opt-on TTL 2.7×, 400 mm working distance; orangedental, Biberach, Germany).

After this internal tunnel approach, the inner enamel surfaces (located at the depth of the occlusal cavity) of all teeth were dried by means of a compressed air stream (Sprayvit, Teneo; Dentsply Sirona), followed by total etch using 40% phosphoric acid gel (HS Etch Gel; Henry Schein). The etchant was thoroughly removed using an air/water sprayer (Sprayvit, Teneo; Dentsply Sirona; 30 s) after an exposure time of 1 min. Subsequently, the cavity was fully dried with oil-free, compressed air (Sprayvit, Teneo; Dentsply Sirona; 30 s).

Prior to infiltration, the infiltrant (Icon Caries Infiltrant; DMG; 2 drops) was labeled using a green fluorescent dye (0.1 mmol fluorescein isothiocyanate, FITC; Babenberger Apotheke, Vienna, Austria). For exact dispensing, 10 μl of FITC were pipetted (Research plus–PhysioCare Concept; Eppendorf, Hamburg, Germany; Pipette tips epT.I.P.S, 200 μl; Eppendorf) into mixing pads (#9008146, HS-Mixing palette; Henry Schein). After 20 min, the alcohol was fully evaporated; then, the dye was mixed with 2 drops of the resin infiltrant using a micro-brush (Microbrush Plus, superfine white, Ø 1 mm; Microbrush International, Grafton, WI, USA). Subsequently, the FITC-labeled infiltrant was carefully applied onto the demineralized inner enamel for 3 min, in each case using a new micro-brush. The resin infiltrant was light-cured (by assuring a 3-mm distance; Mini LED Curing Light, >1,250 mW/cm^2^; Satelec Acteon, Mérignac, France) via the prepared cavity for 40 s, and the resin infiltration procedure was repeated once (1 min), followed by a polymerization for another 40 s. Finally, the entire cavity was filled up with flowable, light-curing composite resin (G-Premio Bond; GC Europe, Leuven, Belgium; and G-ӕnial Flo X shade *A3*; GC Europe), which finally was polymerized for 40 s (see flowchart, Fig 1).

### External etching and drying process

All outer surfaces of the proximal lesions were etched with hydrochloric acid gel (15% HCl, Icon-Etch; DMG; 2 min). Subsequently, the etchant was completely removed using an air/water sprayer (Sprayvit, Teneo; Dentsply Sirona) for 30 s; the teeth were then air-dried for another 30 s (Sprayvit, Teneo; Dentsply Sirona). Afterwards, a complete draining by means of ethanol (99%, Icon-Dry; DMG; 30 s) and oil-free, compressed air (30 s) followed.

### External infiltration of Group 1

All teeth of Group 1 (12 premolars/12 molars; n = 24; ICDAS code 2: n = 12, ICDAS code 3: n = 12) were externally infiltrated (Icon Caries Infiltrant; DMG) according to the recommendations as given by the manufacturer. The resin infiltrant for Group 1 was labeled with a red fluorescent dye (0.1 mmol rhodamine B isothiocyanate, RITC; Babenberger Apotheke). After infiltrating the proximal lesions for 3 min, surpluses were removed by means of dental floss (Oral-B Superfloss; Procter & Gamble, Schwalbach, Germany) without using the spongy floss part, and foam pellets (#1, Ø 4 mm; Henry Schein). Finally, the infiltrated lesions were light-cured for 40 s (Mini LED Curing Light, >1,250 mW/cm^2^; Satelec Acteon). This infiltration procedure was repeated once (1 min infiltration, 40 s polymerization time).

### External infiltration of Group 2

In line with Group 1, the resin infiltrant for Group 2 (12 premolars/12 molars; n = 24; ICDAS code 2: n = 12, ICDAS code 3: n = 12) was first labeled with a red fluorescent dye (0.1 mmol rhodamine B isothiocyanate, RITC; Babenberger Apotheke); subsequently, the resin was hand-mixed with AgNP (20 nm particle size [48], 5.5 wt%; Ionic Liquids Technologies, Heilbronn, Germany). Infiltration procedure, removal of surpluses, re-infiltration, and polymerization were performed in analogy to Group 1 (see flowchart, Fig 1).

### Preparation of the specimens and microscopic evaluation

Prior to the microscopic examinations, either the buccal or the lingual surface of each tooth was partially ground using a grinder/polisher (MetaServ 250 with Vector Power Head; Buehler, Lake Bluff, IL, USA; CarbiMet, silicon carbide [SiC] grinding paper, P 320; Buehler); this was done parallel to the tooth axis under constant water cooling. Then, the specimens were gently dried with a soft paper towel, and fixed with the grounded surface downside (Sekundenkleber; UHU, Bühl, Germany) on a previously roughened (using SiC abrasive paper (Matador, P 220; Starcke, Melle, Germany)) glass microscopic slide (#190501, 28 × 48 × 1 mm, Menzel-Gläser; Thermo Fisher Scientific, Waltham, MA, USA).

Subsequently, the teeth were cut (IsoMet 1000; Buehler; and IsoMet 15HC metal matrix, #11–4246, 0.5 mm, precision sectioning blade; Buehler) in the mesio-distal direction next to the most carious aspect of the lesion (as previously defined by the laser detection device, see above) under permanent water cooling. Next, each cut tooth was hand-polished (MetaServ 250/Vector Power Head; Buehler; CarbiMet, SiC grinding paper, P 1,200; Buehler; Buehler-Met II, SiC grinding paper, P 2,500; Buehler; MicroCut, SiC grinding paper, P 4,000; Buehler) until the most demineralized area was visible. To ensure that the desired depth range was reached, a light microscope (Nikon SMZ645/Nikon G-AL 1.5×; Nikon, Tokyo, Japan) and magnifying glasses (opt-on TTL 2.7×, 400 mm working distance; orangedental) were used.

After the grinding process, all cut surfaces were cleaned using sodium hypochlorite (NaOCl, 5%; Sigma-Aldrich, Steinheim, Germany; 2 h), to get rid of any smear layer, and to dissolve any organic material potentially occluding the porous lesion volume [57]. Then, the teeth were stored in 50 ml of hydrogen peroxide (30%, Sigma-Aldrich) for another 2 h, to completely dissolve the sodium hypochlorite, and to bleach organic residues remaining in the non-infiltrated enamel pores; this procedure ensured a reduction of the teeth’s auto-fluorescence. All teeth were then immersed into tap water at room temperature (4 h). Finally, all surfaces were dried with absolute alcohol (Merck, Darmstadt, Germany) and coated with a blue fluorescent dye (0.1 mmol Berberine; Babenberger Apotheke) using a micro-brush (Microbrush Plus, superfine white, Ø 1 mm; Microbrush International), to allow for a clear differentiation between the infiltrated and the non-infiltrated areas. After 2 h the colorant was rinsed gently into a basin for 1 min, to remove any surface excess of the fluorescent dye.

Using a confocal laser scanning microscope (CLSM; TCS SP8 DMi8; Leica Microsystems, Wetzlar, Germany) and a 10× objective (HC PL Fluotar 10×/0.3 numerical aperture, dry; Leica Microsystems) the lesions of all teeth were captured. The format of the images was individually chosen depending on the size of each lesion. To obtain two-dimensional images, the xy-scan modus was combined with separate extinction settings (561 nm–red/RITC; 520 nm–green/FITC; 405 nm–blue/Berberine). For image analysis, an open source software tool (GIMP 2.8.16 GNU Image Manipulation Program; https://www.gimp.org) was used. By means of GIMP’s ‘Paths Tool’, each lesion was outlined. Then, with each tooth 10 measuring points were randomly selected within the outlined lesion of the red, the green, and the blue image using the ‘Color Picker’ tool to evaluate the individual fluorescence values for FITC, RITC, and Berberine. Subsequently, the thresholds for RITC, FITC, and Berberine were adjusted excluding the above-mentioned tooth-specific individual fluorescence values to use the ‘Histogram Dialog’ tool aiming to obtain the number of pixels within the outlined area of demineralized enamel and of the infiltrated demineralized enamel areas as well as the percentage values of the infiltration areas.

### Measured variables and statistical analysis

Raw data (recorded in pixels) were entered into Excel sheets (Microsoft, Redmond, WA, USA). Both the total lesion sizes of enamel (TLS_Enamel_) and the infiltrated lesion areas (ILA_Enamel_) were now fractionized with regard to the used fluorescents (FITC, RITC, or Berberine), and the percentages of proportions of infiltrated lesion areas (% ILA_Enamel_) were computed (% ILA_Enamel_ = ILA_Enamel_ × 100 ÷ TLS_Enamel_); the same was calculated with the non-infiltrated lesion areas. All statistical analyses were performed by means of a statistical software package (IBM SPSS Statistics 25; IBM Analytics, Armonk, NY, USA), including calculation of means, medians, standard deviations, and quartiles. Homoscedasticity (equality of variances) of the relevant parameters (total lesion size with regard to either tooth type or ICDAS code) was assessed by means of the Levene’s test, and the latter was used to test for possible differences in sample variances of the various infiltrated lesion areas (ILA_Enamel_ with regard to FITC, RITC, or Berberine; with or without the use of AgNP). After testing for normal distribution according to the K-S-test (Lilliefors approximation), subgroup comparisons were analyzed using independent *t*-test statistics, and equalities of means were assessed. With all statistical comparisons, significance levels of 5% (α = 0.05) indicated significant differences that were unlikely to have arisen by chance.

## Results

During the preparation procedures for histological validation, 5 teeth were lost due to irreparable damage, thus resulting in 21 premolars and 22 molars (ICDAS code 2: n = 21, ICDAS code 3: n = 22) to be processed. According to Levene’s test, the homoscedasticity could not be refused (minimum p-value was 0.224), thus revealing comparable total enamel lesion sizes, regardless of tooth types or ICDAS codes. Therefore, the various subgroups could be merged, now considering conventional infiltration (Group 1; n = 19) or infiltration with additional use of AgNP (Group 2; n = 24) as the remaining subgroups.

The internal infiltration procedure (using FITC as fluorescent marker) resulted in substantially infiltrated lesion areas, with a mean of some 50% of the TLS_Enamel_ being infiltrated after bleaching of possibly fluorescent organic remnants. The *t*-test statistics did not reveal any significant differences between Group 1 and Group 2 (p = 0.838; see Table 1). Regarding the external infiltration (using RITC), both groups displayed infiltrated lesion areas with a mean of some 20% of the TLS_Enamel_, again without any significant differences (p = 0.109; Table 1); the outer lesion areas obviously were totally occluded by the resinous infiltrant.

**Table 1:** Infiltrated lesion areas (in %) in relation to total enamel Lesions.

	**ILA**_**Enamel**_ **(Internal Infiltration, FITC), % of TLS**_**Enamel**_					
	**Mean**	**SD**	**Median**	**q1**	**q3**	**Sig**.
**Group 1** Infiltration without AgNP	54.74	16.26	56.00	42.00	63.00	p = 0.838
**Group 2** Infiltration with AgNP	55.88	19.21	58.00	41.25	70.50	
	**ILA**_**Enamel**_ **(External Infiltration, RITC), % of TLS**_**Enamel**_					
	**Mean**	**SD**	**Median**	**q1**	**q3**	**Sig**.
**Group 1** Infiltration without AgNP	18.84	8.30	16.00	12.00	24.00	p = 0.109
**Group 2** Infiltration with AgNP	26.29	20.10	20.50	14.25	33.50	
	**Non-infiltrated Lesion Area (Berberine), % of TLS**_**Enamel**_					
	**Mean**	**SD**	**Median**	**q1**	**q3**	**Sig**.
**Group 1** Infiltration without AgNP	71.95	12.20	76.00	61.00	82.00	p = 0.965
**Group 2** Infiltration with AgNP	72.13	14.08	72.00	61.25	85.75	
	**ILA**_**Enamel**_ **(Total Infiltration, FITC + RITC), % of TLS**_**Enamel**_					
	**Mean**	**SD**	**Median**	**q1**	**q3**	**Sig**.
**Group 1** Infiltration without AgNP	73.58	22.11	76.00	54.00	86.00	p = 0.381
**Group 2** Infiltration with AgNP	82.17	37.32	77.00	56.50	98.00	

ILA_Enamel_, infiltrated lesion area of enamel; TLS_Enamel_, total enamel lesion size of enamel; statistical parameters (means, standard deviation [SD], medians, first [q1] and third [q3] quartiles) as well as exact p values [Sig.] are given for both the internally and the externally infiltrated areas, along with the non-infiltrated lesions areas and the totally infiltrated lesion areas.

**Fig 3:**
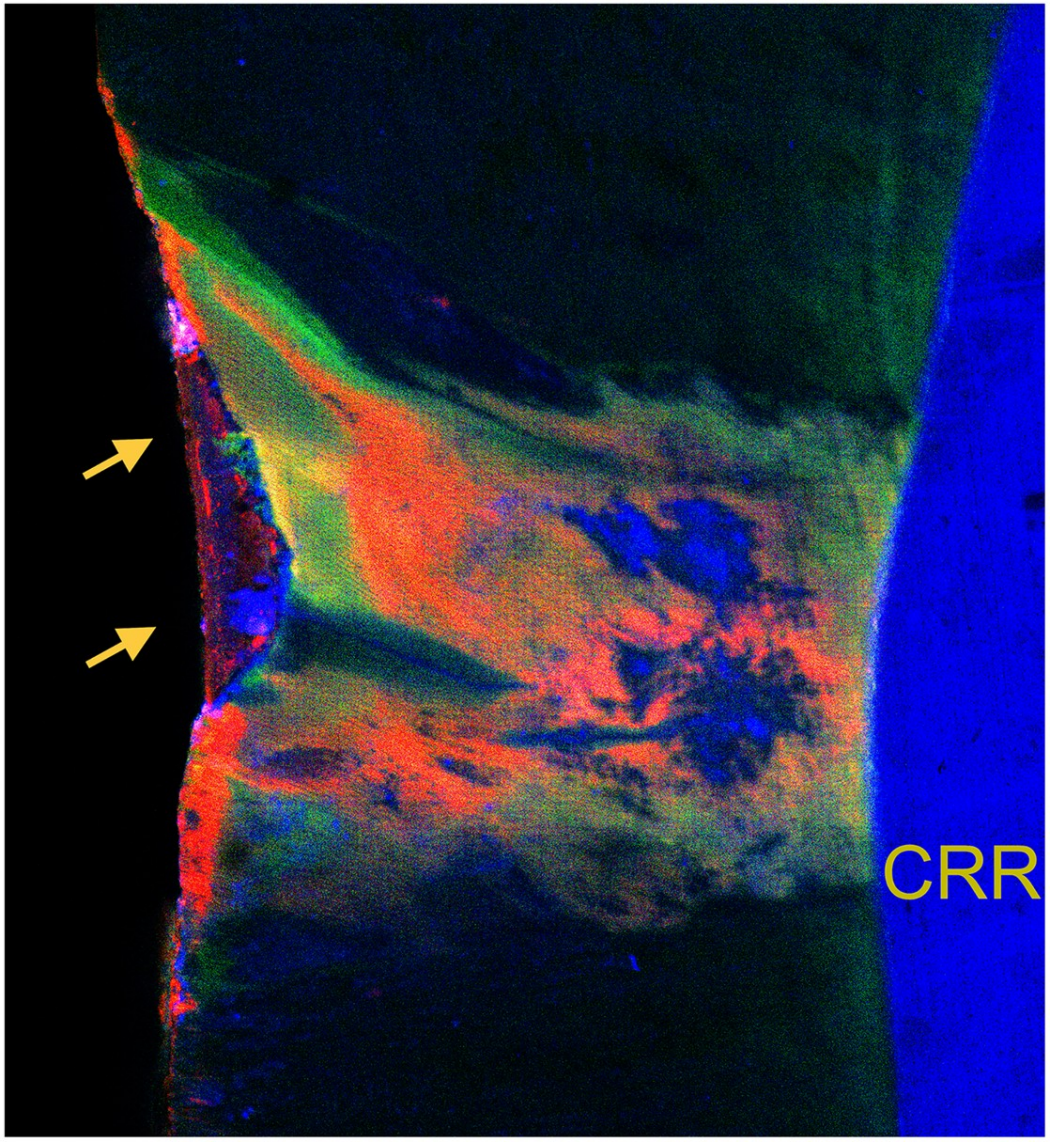
Merged confocal laser scanning micrograph. Micrograph (10× magnification) corresponding to the specimen known from Fig 2, and revealing the deep and partially inhomogeneous penetration of the resin infiltrant into the lesion body, visualized by the fluorescently labeled infiltrant resin (internal infiltration with FITC-labeled resin, green; external infiltration with RITC-labeled resin, red; Berberine filling the porous volume, blue), at the same time depicting interdiffusion zones of internal and external infiltration. Note the adhesive seal of the restoration (right), and the partially filled surface damage of the lesion (left part, see arrows). [CRR, composite resin restoration; FITC, fluorescein isothiocyanate; RITC, rhodamine B isothiocyanate.].

None of the lesions of both groups was completely infiltrated; a representative confocal laser scanning micrograph is provided with Fig 3, depicting the non-infiltrated areas in blue (Berberine). The statistical analysis revealed that both groups did not differ significantly with respect to the percentage distribution of non-infiltrated areas (p = 0.965; Table 1). This could be confirmed by cross-checking with the total infiltration areas (internal plus external infiltration, FITC plus RITC); here, again, no significant differences could be assessed by the statistical analysis either (p = 0.381; Table 1).

## Discussion

With the present study, we introduce a refined treatment concept for proximal lesions clearly extending into dentin. From recent investigations, it is known that dentin subjacent to natural proximal enamel caries is not simply sclerotic (as has been presumed sometimes); instead, these dentin portions have been shown to be demineralized in wide parts [58], thus substantially reducing mechanical resistance, and additionally jeopardizing the integrity of the overlying (and likewise weakened) enamel. Very comparable observations have been reported for occlusal caries lesions reaching to the underlying dentin [59]. Therefore, removal of demineralized dentin (and replacement by adequate restorative materials [60]) should be an appropriate treatment option with these lesions.

This procedure would seem accompanied by a bacterial eradication. No doubt, microbial infection of dentin must be assumed with (micro-)cavitated proximal lesions [34, 61], but the level of infection at the cervical cavity floor of proximal lesions can be greatly reduced, both with conventional and with tunnel cavity preparations [62], thus confirming that removal of soft and infected dentin is effective when pursuing a positive patient outcome. Concerning the conventional infiltration therapy, it must be emphasized that due to the barrier created by an external resin infiltration, resistance to external acids will be increased [5, 23] (even if the infiltrated surface is not completely impervious to a new cariogenic challenge [25, 26]), and outside microorganisms will be expelled. From previous investigations it is known, however, that bacteria can be found within enamel lesions at an early stage of caries development, even with lesions revealing macroscopically intact surfaces [63]. Since bacteria control of deeper intra-lesional aspects has not been investigated up to now, at best, some positive indirect conclusions by analogy from studies referring to sealing of occlusal caries would seem permissible [64], and this would suggest that it is not necessary to remove all carious dentin prior to placing the restoration; over time, sealing of carious dentin obviously will result in lower (time- and material-specific [65]) levels of infection if compared to the traditional concepts aiming at complete dentin caries removal [66]. Certainly, additional research to elucidate the fate of microorganisms hemmed by the external resin infiltration of proximal lesions is required.

Next to the mechanistic and biologic thoughts given above, it should be underlined that the demineralized dentin portion subjacent to a proximal enamel lesion must be expected to provide enlarged pathways for dentinal fluids to penetrate into the proximal caries [58], thus hampering the latter from complete drying, competing with the low-viscosity infiltrant resin, and rendering a complete infiltration impossible from a clinical point of view. This would explain that external resin infiltration alone probably will not be able to completely permeate into the full-thickness of a lesion [7], all the more so as the percentage penetrations of the resin with proximal caries considerably exceeding the enamel-dentin border is notoriously lower than with lesions exclusively restricted to enamel [67]. Thus, even if the technique has been rated effective in arresting the progression of non-cavitated proximal caries involved in the enamel-dentin junction, it would not seem astonishing that for proximal caries clearly exceeding the enamel-dentin border, the therapeutic efficacy of solely external resin infiltration clearly is limited. This has been shown with recent studies focusing on deeper proximal caries [7, 10], thus shedding some ambiguous light on possible survival of trapped microbiota (and their acid production as well as, beyond, their proteolytic and hydrolytic activities). Consequently, this would suggest that due consideration is mandatory with treatment decisions on proximal caries involving the vicinal dentin.

The internationally accepted “gold standard threshold” for minimally invasive operative interventions currently refers to proximal lesions radiographically extending beyond the outer third of dentin (with recommendations aiming at predominantly saucer-shaped preparations as the favored cavity design) [68]. This mainly is owed to the fact of increasing cavitation probabilities of the outer enamel surface [33, 34, 69], and these breakdowns, in turn, correspond to impaired biofilm removal and decreasing remineralization capabilities (even with patients showing normal salivary function [70]); moreover, it is well-known that lesions extending clearly beyond the enamel-dentin border (> 0.5 mm) are most likely to progress within a period of 3 years [71], and comparable deteriorations have been reported with increasing ICDAS severities [36]. Therefore, in accordance with the current study set-up, temporary separation of teeth to inspect the proximal surface integrity [72] and DIAGNOcam readings to scrutinize lesion severities [56, 73] would seem advisable prior to any treatment decision.

With proximal caries extending into dentin, modified partial [74] or internal [30] tunnel restoration techniques (both actually representing a Class I cavities), allowing for an internal and external resin infiltration have been suggested recently [32]; it should be emphasized that this concept of double-sided resin infiltration would seem consistent with the prevailing recommendations on treatment decisions related to surgical intervention. Indeed, the tunnel preparation approach (even if challenging with regard to caries removal [74]) dispenses with full surgical intervention as the last resort [14], and aims at a complete preservation of the proximal enamel, including the previously demineralized lesion, now reinforced by means of the double-sided infiltration [32]. Although the non-filled polymerized infiltrant resin itself reveals a low microhardness (even after accelerated aging) [37], it is noteworthy that the reported increase of surface microhardness of infiltrated enamel lesions [17–22, 25] is comparably high; this obviously is due to a uniform complex composed of triethylene glycol dimethacrylate (TEGDMA) and hydroxyapatite, and this interaction with crystals results in improved mechanical strengths [22] and aesthetic appearance [2, 23].

With an internal penetration depth obstructing more than 50% of the TLS_Enamel_ (see Table 1), the current study indeed revealed a considerable portion of internally occluded carious enamel after a conventional total etch procedure using phosphoric acid, and this clearly outperformed our recently published outcome of internal infiltration [32]. Indeed, internal infiltration obviously should be the first step, and occlusion of the tiny pores might even be enhanced by applying active pressure with the aid of packable composite resins prior to polymerization [75]. The infiltrant resin used in the present investigation (Icon Caries Infiltrant; DMG) has been shown to be compatible to generally utilized adhesive restorations [32, 76], and does not impair the shear bond strengths to dentin [77], while adhesion to sound [78–80] or demineralized [40, 78, 80, 81] enamel even was increased (if compared to other adhesive systems); moreover, tensile bond strength testing was accompanied by a high portion of mixed (cohesive in enamel) failures [80], thus indicating a strong and reliable enamel hybrid layer composed of resin tags enveloping the enamel crystallites [82]. These aspects obviously indicate that final composite resin restorations can be bonded adhesively to the resin-infiltrated hard substances, and this should result in stable repairs, thus complying with the concepts of minimum intervention dentistry [1, 2], and solving (or at least minimizing) some of the clinical problems discussed above.

In the past, tunnel restorations often have been rated with some reservation, and several reasons did account for this conservative or reticent attitude. First, and this is considered important with respect to the current study, the majority of the previous studies investigating the tunnel technique used glass ionomers as restorative materials. With regard to adhesive effects and reinforcement, these must be classified as inferior if compared to composite resins [83]. Consequently, the latter have shown more promising results, with positive laboratory evaluations [83–85], and with high clinical success rates (but with short observation periods of up to 2 years only) [31, 86]. Second, and this should be a consequence of the material-related aspects, former failures were mainly due to fractures of the marginal ridges [87]. Thus, to maintain tooth strength and integrity, an intact marginal ridge should be preserved whenever possible [88]. Therefore, when complying with some preparation guidelines (height of marginal ridge [83–85], width of marginal ridge [83, 89–91], and cavity size [92]), internal tunnels (Class I cavities) should be a feasible treatment option [30]. Additionally, adhesive composite resins exerting strengthening properties like resistance to fracture, failure mode, or stress distribution [93] similar to the unaltered tooth (with a 0.87 relative stiffness [94]) should have a positive effect on fracture resistance, even with reduced marginal ridge dimensions [83, 89]. Finally, further deterioration of the enamel lesion has been blamed to be responsible for the frequent failures of tunnel restorations [95], so both hampering the undermining lesion progress and stabilizing the demineralized lesion by creating an enamel hybrid layer [82] using the internal/external resin infiltration approach should help to maintain tooth rigidity. Nevertheless, while the thoughts given above would seem plausible at a first glance, confirmation of an extensive compatibility of timely adhesive restorative materials to sound dentin (with its functional aspects of elasticity, rigidity, and toughness) would seem mandatory [90], and the clinical efficacy of the approach presented with the current paper has not been corroborated up to now.

In the present study, proximal surfaces revealing initial caries lesions (ICDAS 2) or those considered at the transition to moderate lesions (ICDAS 3) [14, 15] have been used; these lesions can be considered as fast progressing ones, and a therapeutic intervention has been recommended sooner rather than later [36]. Along with the radiologic diagnosis, we used the DIAGNOcam (KaVo) readings for the final tooth selection, since near-infrared transillumination clearly enables differentiation of lesions limited to enamel from those reaching the dentin. It should be kept in mind that bitewing radiographs tend to underestimate lesion depths [55, 96], even if combined with visual inspection [56]; instead, the use of near-infrared transillumination does disclose the true lesions extent more accurately [73], and, although not considered our primary aim, this has been corroborated by the current study.

Additionally, treatment needs (and in particular the localization of the sectioning area) were assured by means of DIAGNOdent pen (KaVo) with the present set-up. From a histological point of view, enamel lesion sizes proved to be comparable, and, as expected [36], all lesions used in the current investigation had reached the dentin, and turned out to be accessible both for internal and for external infiltration, while this was neither influenced by tooth types nor by ICDAS codes. Thus, clustering of study subsets with respect to the use of AgNP was possible, and the statistical evaluation did not reveal any differences between the both groups, neither regarding the external infiltration nor with reference to the combination of internal and external infiltration. Moreover, all lesions revealed non-infiltrated areas to some extent; concerning this matter, no significant differences could be revealed as well (see Table 1). Within the scope of an overall view, adding AgNP to the infiltrant resin did not affect the infiltration ability of the latter. Consequently, under the limitations of the present *ex vivo* study, the null hypothesis was not rejected.

With the current set-up, we assessed infiltrated lesion areas by evaluating the microscopic images in due consideration of the respective fluorescence modes (and not by analyzing the merged overlays, compare Fig 3). It should be mentioned, that the merged microscopic images showed overlaps of the different fluorescents (FITC, RITC, Berberine). Due to this fact, it can be concluded that the individual pores of the respective lesions having been stained either red (RITC) or green (FITC) have been incompletely infiltrated; presenting some blending with the blue Berberine, this would suggest that the latter was able to penetrate into residual pores previously occluded by organic compounds or trapped air [57], and obviously not completely filled by the polymerized low-viscosity infiltrant resin. However, to the best of our knowledge, we did not find any further information regarding this aspect in the literature, and more research to evaluate the exact distribution of the respective infiltrant portions would seem mandatory.

Recent studies have revealed that organic matter deposited in the porous lesion volume will hamper both remineralization and resinous infiltration of subsurface lesions [57], and removal of this organic debris has been considered pivotal for successful treatment [39]. Therefore, prior to infiltration, the outer surfaces had been deproteinized by means of sodium hypochlorite (5%; 20 min), as has been recommended previously [32, 39, 97]. This (unusually) prolonged cleaning procedure aimed to dissolve organic surface remnants possibly occluding the tiny lesion pores serving as entrance and pathways for the low-viscosity infiltrant resin, and also removed extrinsic deposits possibly covering the micro-cavitations [53], thus appropriately preparing the lesion surface for hydrochloric acid etching [39]. The latter approach will lead to erosive enamel loss [98] and will additionally roughen the surfaces (in particular those with advanced ICDAS codes) [41], whereas the intentionally non-polished infiltrant resin itself obviously is not completely able to smooth out the surface irregularities to an acceptable clinical level (and not comparable to sound enamel) [18–22, 25, 39]; this roughness even should further deteriorate after aging [18]. Representing bi-functional methacrylate monomers, the non-filled diluent TEGDMA component of the infiltrant resin will be inhibited from complete polymerization by free radical scavengers such as oxygen, and loss of this oxygen-inhibited layer might contribute to an increasing roughness with time. Therefore, while finishing procedures will not improve the quality of these rough surfaces [41], bacterial colonization will not be impeded by the infiltrant resin or its main components [17, 19, 42, 99].

While TEGDMA leached from fresh or undercured resin initially (up to 24 h) reduced biofilm metabolic activity (but not biomass) [99], polymerized TEGDMA is prone to water and/or ethanol sorption leading to monomer hydrolysis and fractures of ester bonds [37], and this effect is higher than with other widespread resins [100]. Due to internal stress caused by thermal expansion and contraction, these volumetric changes might result in surface microcracks and microfissures (thus providing entrance for fluids and enzymes), as has been recently reported with resin-infiltrated enamel lesions [18]. Salivary [101] and microbial [102] esterase activities (cholesterol esterase, pseudo-cholinesterase) have revealed hydrolysis of TEGDMA, and in particular the matrix biodegradation promoted by bacterial esterases seems to be responsible for the increased composite surface roughness upon biofilm exposures [103]. It should be noted that hydrolytic degradation (by firmly bound water in the enamel pores and water from saliva) yields a hydrophilic product, triethylene glycol (TEG), which has been reported to stimulate growth and pathogenicity of *Strep*. *mutans* and *Strep*. *salivarius* [104, 105], thus again leading to increased biofilm accumulation. With the aspects given above, the hydrophilic infiltrant resin is considered a low-level resistant material with progressively reduced mechanical properties [37], judged vulnerable to deterioration in the oral environment [23], and revealing a time-dependent reduction of microhardness [106]. Whereas the otherwise non-controversial and favorable characteristics of TEGDMA as an infiltrant resin should not to be pilloried, the context presented above would seem to elucidate the limited efficacy of resin infiltration with deeper proximal caries lesions [7, 10].

Undoubtedly, strategies including optimized oral hygiene and prudent dietary control are considered paramount to control caries, as has been shown previously with long-existing white spot lesions located on smooth surfaces, which proved to be stable under the conditions of adequate mouth hygiene [107]. Initial proximal lesions, however, are neither clearly visible nor adequately assessable in the majority of cases, and this in particular comes true with progressed caries revealing surface (micro-)breakdown [33, 34], which renders cleansing impossible (compare Fig 2A). Thus, the concept developed for the current study was to combine the infiltrant with an antibacterial agent, such as nanosilver; this additive should safeguard the infiltrated (but still rough) surface areas from *de novo* microbial colonization and subsequent biodegradation.

It should be kept in mind that proximal caries lesions and conventional restorative treatment options will constitute microbial proximal invasions, considered suspicious of negatively affecting outer natural tooth surfaces, and responsible for long-term occlusal and periodontal sequelae [108]. The internal tunnel approach combining internal and external (double-sided) infiltration of the enamel lesions as studied in the current investigation should help to overcome the problems of proximal lesions progressed into dentin and hitherto not designated to the external (single-sided) infiltration approach, thus sustaining the tooth’s proximal outline, and delaying the reparative treatment cycle usually associated with conventional Class II preparations (with drawbacks like marginal excess or gaps of filling materials, poor proximal polish of restorations, mal-contouring, complex refurbishment of proximal contact areas, accidental injuries of neighboring teeth, and/or impaired periodontal health [2, 32]).

As with other restorative treatments, the primary aim of infiltration therapy is to facilitate biofilm control. In the present study, external infiltration of the infiltrant resin was not hampered by the nanosilver particles, and reached percentage values of up to some 25% of the TLS_Enamel_. While this was considered comparable to our previous study [32], AgNP concentrated outside the lesion, and enriched in locations of tiny surface disintegration. The latter effect was comparable to recent observations using the same infiltrant resin along with pre-polymerized methacrylate-based nanofillers [109], and clearly revealed a segregation of the components during the infiltration process (see Fig 3). This targeted accumulation is considered advantageous; it should be borne in mind that a reduced biofilm mass would render the contact-killing mechanisms of nanosilver particles effective. Interestingly enough, AgNP have been described to significantly suppress the growth of *Strep*. *mutans* and *Lactobacillus* with down to 1% by weight added to commercial composite resins [48, 49], thus confirming the anticariogenic outcome observed with high AgNP concentrations of up to 7 wt% [110, 111].

Hence, in accordance with those previous studies [110, 111], we have chosen 5.5 wt% of AgNP, to vet any possible detrimental effects of the nanoparticles on penetration ability of the infiltrant resin. It is known that, next to the long-term bactericidal activity and the decreased lactic acid production driven by the nanosilver [50, 112, 113], AgNP increased the surface hydrophobicity of composite resins, even with low mass fractions of down to 0.3 wt% [114]. Additionally, it is worth mentioning that with these low concentrations, mechanical characteristics (like flexural strength, or elastic modulus) of the composites matched those of commercial products [112, 113], even though some reports have indicated an influence on polymerization, thus increasing the amount of elutable monomers [115]. This would seem to indicate that the addition of AgNP should not alter the properties of the well-polymerized infiltrant resin. In total, the conceptual shift presented with the current proof-of-concept study should take control over lesion activity of proximal caries lesions extending into dentin (and already revealing minor surface breakdown); along with a monitored preventive regimen, this combined concept of managing the biofilm-mediated and diet-modulated, multifactorial disease called dental caries would seem successful, even over the long term [14, 60]. Though the aspects outlined above have not been evaluated up to now, the underlying background rationale would seem justified to increase dental health, and further studies undoubtedly would seem warranted.

## Conclusion

From the current *ex vivo* investigation, it can be concluded that the ability of the studied infiltrant resin to infiltrate into non- and micro-cavitated proximal enamel caries progressing into dentin will not be negatively impacted by the addition of AgNP. Based on these observations, it would seem reasonable to deduce that the antibacterial effects of AgNP-containing infiltrant resin should hamper the re-formation of microbial biofilms, thus increasing the durability of the infiltrated lesion without compromising its mechanical properties, and impeding the development of recurrent or secondary caries. With the presented internal tunnel approach, the combination of internal and external resin infiltration should result in an increased stabilization of the demineralized enamel and should prevent any sacrifice of sound enamel, thus fostering the transition of dentistry to a minimally invasive and disease-based discipline, and reducing the fatal and avoidable cycle of re-dentistry.

## Supporting information

S1 File (PDF)

## References

[1] FDI World Dental Federation. FDI policy statement on Minimal Intervention Dentistry (MID) for managing dental caries: Adopted by the General Assembly: September 2016, Poznan, Poland. Int Dent J. 2017; 67(1): 6–7. 10.1111/idj.12308 28194784 PMC9376679

[2] Kielbassa AM, Müller J, Gernhardt CR. Closing the gap between oral hygiene and minimally invasive dentistry: A review on the resin infiltration technique of incipient (proximal) enamel lesions. Quintessence Int. 2009; 40(8): 663–681. 19639091

[3] Kielbassa AM, Ulrich I, Treven L, Mueller J. An updated review on the resin infiltration technique on incipient proximal enamel lesions. Med Evol. 2010; 16(4): 3–15. 10.13140/RG.2.2.36646.37443

[4] Paris S, Meyer-Lueckel H, Cölfen H, Kielbassa AM. Resin infiltration of artificial enamel caries lesions with experimental light curing resins. Dent Mater J. 2007; 26(4): 582–588. 10.4012/dmj.26.582 17886464

[5] Enan ET, Aref NS, Hammad SM. Resistance of resin-infiltrated enamel to surface changes in response to acidic challenge. J Esthet Restor Dent. 2019; 31(4): 353–358. 10.1111/jerd.12471 30893512

[6] Chatzimarkou S, Koletsi D, Kavvadia K. The effect of resin infiltration on proximal caries lesions in primary and permanent teeth. A systematic review and meta-analysis of clinical trials. J Dent. 2018; 77: 8–17. 10.1016/j.jdent.2018.08.004 30092238

[7] Liang Y, Deng Z, Dai X, Tian J, Zhao W. Micro-invasive interventions for managing non-cavitated proximal caries of different depths: A systematic review and meta-analysis. Clin Oral Investig. 2018; 22(8): 2675–2684. 10.1007/s00784-018-2605-9 30238416

[8] Çağlar E, Kuşçu ÖÖ, Hysi D. Four year evaluation of proximal resin infiltration in adolescents. Acta Stomatol Croat. 2015; 49(4): 304–308 27688414 10.15644/asc49/4/5 PMC4945337

[9] Urquhart O, Tampi MP, Pilcher L, Slayton RL, Araujo MWB, Fontana M, et al Nonrestorative treatments for caries: Systematic review and network meta-analysis. J Dent Res. 2019; 98(1): 14–26. 10.1177/0022034518800014 30290130 PMC6304695

[10] Peters MC, Hopkins AR Jr., Zhu L, Yu Q. Efficacy of proximal resin infiltration on caries inhibition: Results from a 3-year randomized controlled clinical trial. J Dent Res. 2019; 98(13): 1497–1502. 10.1177/0022034519876853 31526071

[11] Peters MC, Hopkins AR Jr, Yu Q. Resin infiltration: An effective adjunct strategy for managing high caries risk–a within-person randomized controlled clinical trial. J Dent. 2018; 79: 24–30. 10.1016/j.jdent.2018.09.005 30227152

[12] Quock RL, Patel SA, Falcao FA, Barros JA. Is a drill-less dental filling possible? Med Hypotheses. 2011; 77(3): 315–317. 10.1016/j.mehy.2011.05.002 21600700

[13] Ntovas P, Rahiotis C. A clinical guideline for caries infiltration of proximal enamel lesions with resins. Br Dent J. 2018; 225(4): 299–304. 10.1038/sj.bdj.2018.647 30141501

[14] Ismail AI, Pitts NB, Tellez M, Banerjee A, Deery C, Douglas G, et al The International Caries Classification and Management System (ICCMS^™^). An example of a caries management pathway. BMC Oral Health. 2015; 15(Suppl 1): S9 10.1186/1472-6831-15-S1-S9 26391116 PMC4580843

[15] Martignon S, Pitts NB, Goffin G, Mazevet M, Douglas GVA, Newton JT, et al CariesCare practice guide: Consensus on evidence into practice. Br Dent J. 2019; 227(5): 353–362. 10.1038/s41415-019-0678-8 31520031

[16] Kielbassa AM, Wrbas KT, Schulte-Mönting J, Hellwig E. Correlation of transversal microradiography and microhardness on in situ-induced demineralization in irradiated and nonirradiated human dental enamel. Arch Oral Biol. 1999; 44(3): 243–251. 10.1016/s0003-9969(98)00123-x 10217515

[17] Aziznezhad M, Alaghemand H, Shahande Z, Pasdar N, Bijani A, Eslami A, et al Comparison of the effect of resin infiltrant, fluoride varnish, and nano-hydroxy apatite paste on surface hardness and *Streptococcus mutans* adhesion to artificial enamel lesions. Electron Physician. 2017; 9(3): 3934–3942. 10.19082/3934 28461867 PMC5407225

[18] Zhao X, Ren YF. Surface properties and color stability of resin-infiltrated enamel lesions. Oper Dent. 2016; 41(6): 617–626. 10.2341/15-319-L 27589273

[19] Arslan S, Zorba YO, Atalay MA, Özcan S, Demirbuğa S, Pala K, et al Effect of resin infiltration on enamel surface properties and *Streptococcus mutans* adhesion to artificial enamel lesions. Dent Mater J. 2015; 34(1): 25–30. 10.4012/dmj.2014-078 25748455

[20] Yazkan B, Ermiş RB. Effect of resin infiltration and microabrasion on the microhardness, surface roughness and morphology of incipient carious lesions. Acta Odontol Scand. 2018; 76(7): 473–481. 10.1080/00016357.2018.1437217 29447057

[21] Gürdoğan EB, Özdemir-Özenen D, Sandallı N. Evaluation of surface roughness characteristics using Atomic Force Microscopy and inspection of microhardness following resin infiltration with Icon^®^. J Esthet Restor Dent. 2017; 29(3): 201–208. 10.1111/jerd.12279 28333399

[22] Horuztepe SA, Başeren M. Effect of resin infiltration on the color and microhardness of bleached white-spot lesions in bovine enamel (an in vitro study). J Esthet Restor Dent. 2017; 29(5): 378–385. 10.1111/jerd.12308 28568745

[23] de Almendra Freitas MC, Nunes LV, Comar LP, Rios D, Magalhães AC, Honório HM, et al In vitro effect of a resin infiltrant on different artificial caries-like enamel lesions. Arch Oral Biol. 2018; 95: 118–124. 10.1016/j.archoralbio.2018.07.011 30099240

[24] Schneider H, Park KJ, Rueger C, Ziebolz D, Krause F, Haak R. Imaging resin infiltration into non-cavitated carious lesions by optical coherence tomography. J Dent. 2017; 60: 94–98. 10.1016/j.jdent.2017.03.004 28286174

[25] Neres ÉY, Moda MD, Chiba EK, Briso A, Pessan JP, Fagundes TC. Microhardness and roughness of infiltrated white spot lesions submitted to different challenges. Oper Dent. 2017; 42(4): 428–435. 10.2341/16-144-L 28402735

[26] Torres CR, Rosa PC, Ferreira NS, Borges AB. Effect of caries infiltration technique and fluoride therapy on microhardness of enamel carious lesions. Oper Dent. 2012; 37(4): 363–369. 10.2341/11-070-L 22335304

[27] Liu Y, Deng H, Tang L, Zhang Z. Effect of resin infiltration on microhardness of artificial caries lesions [in Chinese]. Chin J Stomatol. 2015; 50(12): 737–741. 26887398

[28] Ei TZ, Shimada Y, Abdou A, Sadr A, Yoshiyama M, Sumi Y, et al Three-dimensional assessment of proximal contact enamel using optical coherence tomography. Dent Mater. 2019; 35(4): e74–e82. 10.1016/j.dental.2019.01.008 30770133

[29] Pandurić V, Mrakužić M, Tarle Z, Katanec D, Prskalo K, Janković B, et al The prevalence of proximal fractures of dental crowns with amalgam fillings. Coll Antropol. 2009; 33(2): 449–453. 19662762

[30] Chu CH, Mei ML, Cheung C, Nalliah RP. Restoring proximal caries lesions conservatively with tunnel restorations. Clin Cosmet Investig Dent. 2013; 5: 43–50. 10.2147/CCIDE.S48567 24019754 PMC3760193

[31] Kinomoto Y, Inoue Y, Ebisu S. A two-year comparison of resin-based composite tunnel and Class II restorations in a randomized controlled trial. Am J Dent. 2004; 17(4): 253–256. 15478486

[32] Kielbassa AM, Ulrich I, Werth VD, Schüller C, Frank W, Schmidl R. External and internal resin infiltration of natural proximal subsurface caries lesions: A valuable enhancement of the internal tunnel restoration. Quintessence Int. 2017; 48(5): 357–368. 10.3290/j.qi.a37799 28294198

[33] Kielbassa AM, Paris S, Lussi A, Meyer-Lueckel H. Evaluation of cavitations in proximal caries lesions at various magnification levels in vitro. J Dent. 2006; 34(10): 817–822. 10.1016/j.jdent.2006.04.001 16730403

[34] Ratledge DK, Kidd EA, Beighton D. A clinical and microbiological study of approximal carious lesions. Part 1: The relationship between cavitation, radiographic lesion depth, the site-specific gingival index and the level of infection of the dentine. Caries Res. 2001; 35(1): 3–7. 10.1159/000047423 11125189

[35] Pitts NB, Ekstrand KR, ICDAS Foundation. International Caries Detection and Assessment System (ICDAS) and its International Caries Classification and Management System (ICCMS)–methods for staging of the caries process and enabling dentists to manage caries. Community Dent Oral Epidemiol. 2013; 41(1): e41–52. 10.1111/cdoe.12025 24916677

[36] Ferreira Zandoná A, Santiago E, Eckert GJ, Katz BP, Pereira de Oliveira S, Capin OR, et al The natural history of dental caries lesions: A 4-year observational study. J Dent Res. 2012; 91(9): 841–846. 10.1177/0022034512455030 22821238 PMC3420396

[37] Chen M, Li J-Z, Zuo Q-L, Liu C, Jiang H, Du M-Q. Accelerated aging effects on color, microhardness and microstructure of ICON resin infiltration. Eur Rev Med Pharmacol Sci. 2019; 23(18): 7722–7731. 10.26355/eurrev_201909_18981 31599398

[38] Paris S, Bitter K, Naumann M, Dörfer CE, Meyer-Lueckel H. Resin infiltration of proximal caries lesions differing in ICDAS codes. Eur J Oral Sci. 2011; 119(2): 182–186. 10.1111/j.1600-0722.2011.00807.x 21410560

[39] Ulrich I, Mueller J, Wolgin M, Frank W, Kielbassa AM. Tridimensional surface roughness analysis after resin infiltration of (deproteinized) natural subsurface carious lesions. Clin Oral Investig. 2015; 19(6): 1473–1483. 10.1007/s00784-014-1372-5 25483122

[40] Arnold WH, Meyer AK, Naumova EA. Surface roughness of initial enamel caries lesions in human teeth after resin infiltration. Open Dent J. 2016; 10: 505–515. 10.2174/1874210601610010505 27733877 PMC5040761

[41] Mueller J, Yang F, Neumann K, Kielbassa AM. Surface tridimensional topography analysis of materials and finishing procedures after resinous infiltration of subsurface bovine enamel lesions. Quintessence Int. 2011; 42(2): 135–147. 21359248

[42] Tawakoli PN, Attin T, Mohn D. Oral biofilm and caries-infiltrant interactions on enamel. J Dent. 2016; 48: 40–45. 10.1016/j.jdent.2016.03.006 26972979

[43] Cameron SJ, Hosseinian F, Willmore WG. A current overview of the biological and cellular effects of nanosilver. Int J Mol Sci. 2018; 19(7): 2030 10.3390/ijms19072030 30002330 PMC6073671

[44] Bapat RA, Chaubal TV, Joshi CP, Bapat PR, Choudhury H, Pandey M, et al An overview of application of silver nanoparticles for biomaterials in dentistry. Mater Sci Eng C Mater Biol Appl. 2018; 91: 881–898. 10.1016/j.msec.2018.05.069 30033323

[45] Noronha VT, Paula AJ, Duran G, Galembeck A, Cogo-Muller K, Franz-Montan M, et al Silver nanoparticles in dentistry. Dent Mater. 2017; 33(10): 1110–1126. 10.1016/j.dental.2017.07.002 28779891

[46] Blöcher S, Frankenberger R, Hellak A, Schauseil M, Roggendorf MJ, Korbmacher-Steiner HM. Effect on enamel shear bond strength of adding microsilver and nanosilver particles to the primer of an orthodontic adhesive. BMC Oral Health. 2015; 15: 42 10.1186/s12903-015-0024-8 25887896 PMC4374300

[47] Sokołowski J, Szynkowska MI, Kleczewska J, Kowalski Z, Sobczak-Kupiec A, Pawlaczyk A, et al Evaluation of resin composites modified with nanogold and nanosilver. Acta Bioeng Biomech. 2014; 16(1): 51–61 24708202

[48] Kasraei S, Sami L, Hendi S, Alikhani MY, Rezaei-Soufi L, Khamverdi Z. Antibacterial properties of composite resins incorporating silver and zinc oxide nanoparticles on *Streptococcus mutans* and *Lactobacillus*. Restor Dent Endod. 2014; 39(2): 109–114. 10.5395/rde.2014.39.2.109 24790923 PMC3978100

[49] Azarsina M, Kasraei S, Yousef-Mashouf R, Dehghani N, Shirinzad M. The antibacterial properties of composite resin containing nanosilver against *Streptococcus mutans* and *Lactobacillus*. J Contemp Dent Pract. 2013; 14(6): 1014–1018. 10.5005/jp-journals-10024-1442 24858742

[50] Cheng L, Zhang K, Zhou CC, Weir MD, Zhou XD, Xu HH. One-year water-ageing of calcium phosphate composite containing nano-silver and quaternary ammonium to inhibit biofilms. Int J Oral Sci. 2016; 8(3): 172–181. 10.1038/ijos.2016.13 27281037 PMC5113087

[51] Li F, Weir MD, Chen J, Xu HH. Comparison of quaternary ammonium-containing with nano-silver-containing adhesive in antibacterial properties and cytotoxicity. Dent Mater. 2013; 29(4): 450–461. 10.1016/j.dental.2013.01.012 23428077 PMC3631003

[52] Central Ethical Review Committee. The use of human body materials for the purposes of medical research (2003). https://www.zentrale-ethikkommission.de/fileadmin/user_upload/downloads/pdf-Ordner/Zeko/Koerpermat-1.pdf (accessed 30 December 2019).

[53] Boston DW, Jefferies SR. Physical effects of non-cavitated explorer probing on enamel smooth-surface carious lesions. Am J Dent. 2015; 28(4): 183–189. 26437497

[54] Young DA, Novy BB, Zeller GG, Hale R, Hart TC, Truelove EL, et al The American Dental Association Caries Classification System for clinical practice: A report of the American Dental Association Council on Scientific Affairs. J Am Dent Assoc. 2015; 146(2): 79–86. 10.1016/j.adaj.2014.11.018 25637205

[55] Abdelaziz M, Krejci I, Perneger T, Feilzer A, Vazquez L. Near infrared transillumination compared with radiography to detect and monitor proximal caries: A clinical retrospective study. J Dent. 2018; 70: 40–45. 10.1016/j.jdent.2017.12.008 29258850

[56] Kühnisch J, Schaefer G, Pitchika V, García-Godoy F, Hickel R. Evaluation of detecting proximal caries in posterior teeth via visual inspection, digital bitewing radiography and near-infrared light transillumination. Am J Dent. 2019; 32(2): 74–80. 31094141

[57] de Holanda Ferreira DA, Aires CP, De Figueiredo RC, de Sousa FB. High amount of organic matter during caries formation reduces remineralization and resin infiltration of enamel caries. Caries Res. 2018; 52(6): 580–587. 10.1159/000488211 29723862

[58] de Barros Pinto L, Lira M, Cavalcanti YW, Dantas ELA, Vieira MLO, de Carvalho GG, et al Natural enamel caries, dentine reactions, dentinal fluid and biofilm. Sci Rep. 2019; 9(1): 2841 10.1038/s41598-019-38684-7 30808878 PMC6391475

[59] Campos SAG, Vieira MLO, de Sousa FB. Correlation between ICDAS and histology: Differences between stereomicroscopy and microradiography with contrast solution as histological techniques. PLoS ONE. 2017; 12(8): e0183432 10.1371/journal.pone.0183432 (correction: PLoS ONE. 2018; 13(1): e0192270. 10.1371/journal.pone.0192270 29377942) 28841688 PMC5571903

[60] Schwendicke F, Frencken JE, Bjørndal L, Maltz M, Manton DJ, Ricketts D, et al Managing carious lesions: Consensus recommendations on carious tissue removal. Adv Dent Res. 2016; 28(2): 58–67. 10.1177/0022034516639271 27099358

[61] Bakhshandeh A, Floriano I, Braga MM, Thorlacius KA, Ekstrand KR. Relationship between depth of approximal caries lesions and presence of bacteria in the dentine in primary and permanent posterior teeth: A radiographic examination with microbiological evaluation. Acta Odontol Scand. 2018; 76(7): 509–514. 10.1080/00016357.2018.1444201 29484911

[62] Ratledge DK, Kidd EA, Beighton D. A clinical and microbiological study of approximal carious lesions. Part 2: Efficacy of caries removal following tunnel and class II cavity preparations. Caries Res. 2001; 35(1): 8–11. 10.1159/000047424 11125190

[63] Seppä L, Alakuijala P, Karvonen I. A scanning electron microscopic study of bacterial penetration of human enamel in incipient caries. Arch Oral Biol. 1985; 30(8): 595–598. 10.1016/0003-9969(85)90078-0 3863557

[64] Oong EM, Griffin SO, Kohn WG, Gooch BF, Caufield PW. The effect of dental sealants on bacteria levels in caries lesions: A review of the evidence. J Am Dent Assoc. 2008; 139(3): 271–278. 10.14219/jada.archive.2008.0156 18310731

[65] Marggraf T, Ganas P, Paris S, Schwendicke F. Bacterial reduction in sealed caries lesions is strain- and material-specific. Sci Rep. 2018; 8(1): 3767 10.1038/s41598-018-21842-8 29491366 PMC5830646

[66] Maltz M, Henz SL, de Oliveira EF, Jardim JJ. Conventional caries removal and sealed caries in permanent teeth: A microbiological evaluation. J Dent. 2012; 40(9): 776–782. 10.1016/j.jdent.2012.05.011 22664566

[67] Liu YH, Ge LH, Zhang ZY, Chi XQ, Hou FC, Chen HZ. An experimental study on the penetration abilities of resin infiltration into proximal caries lesions in primary molars [in Chinese]. Chin J Stomatol. 2012; 47(11): 684–688. 10.3760/cma.j.issn.1002-0098.2012.11.011 23302432

[68] Laske M, Opdam NJ, Bronkhorst EM, Braspenning JC, van der Sanden WJ, Huysmans MC, et al Minimally invasive intervention for primary caries lesions: Are dentists implementing this concept? Caries Res. 2019; 53(2): 204–216. 10.1159/000490626 30107377 PMC6425814

[69] Wenzel A. Radiographic display of carious lesions and cavitation in approximal surfaces: Advantages and drawbacks of conventional and advanced modalities. Acta Odontol Scand. 2014; 72(4): 251–264. 10.3109/00016357.2014.888757 24512205

[70] Tschoppe P, Wolgin M, Pischon N, Kielbassa AM. Etiologic factors of hyposalivation and consequences for oral health. Quintessence Int. 2010; 41(4): 321–333. 20305867

[71] Foster LV. Three year in vivo investigation to determine the progression of approximal primary carious lesions extending into dentine. Br Dent J. 1998; 185(7): 353–357. 10.1038/sj.bdj.4809812 9807919

[72] Urzúa I, Cabello R, Marín P, Ruiz B, Jazanovich D, Mautz C, et al Detection of approximal caries lesions in adults: A cross-sectional study. Oper Dent. 2019 10.2341/17-314-C 31502921

[73] Melo M, Pascual A, Camps I, Ata-Ali F, Ata-Ali J. Combined Near-Infrared Light Transillumination and direct Digital Radiography increases diagnostic in approximal caries. Sci Rep. 2019; 9(1): 14224 10.1038/s41598-019-50850-5 31578410 PMC6775139

[74] Strand GV, Tveit AB. Effectiveness of caries removal by the partial tunnel preparation method. Scand J Dent Res. 1993; 101(5): 270–273. 10.1111/j.1600-0722.1993.tb01118.x 8248727

[75] Ebert J, Frankenberger R, Petschelt A. A novel approach for filling tunnel-prepared teeth with composites of two different consistencies: A case presentation. Quintessence Int. 2012; 43(2): 93–96. 22257869

[76] Kielbassa AM, Ulrich I, Schmidl R, Schüller C, Frank W, Werth VD. Resin infiltration of deproteinised natural occlusal subsurface lesions improves initial quality of fissure sealing. Int J Oral Sci. 2017; 9(2): 117–124. 10.1038/ijos.2017.15 28621326 PMC5518973

[77] Jia L, Stawarczyk B, Schmidlin PR, Attin T, Wiegand A. Influence of caries infiltrant contamination on shear bond strength of different adhesives to dentin. Clin Oral Investig. 2013; 17(2): 643–648. 10.1007/s00784-012-0727-z 22526893

[78] Naidu E, Stawarczyk B, Tawakoli PN, Attin R, Attin T, Wiegand A. Shear bond strength of orthodontic resins after caries infiltrant preconditioning. Angle Orthod. 2013; 83(2): 306–312. 10.2319/052112-409.1 22908947 PMC8793655

[79] Jia L, Stawarczyk B, Schmidlin PR, Attin T, Wiegand A. Effect of caries infiltrant application on shear bond strength of different adhesive systems to sound and demineralized enamel. J Adhes Dent. 2012; 14(6): 569–574. 10.3290/j.jad.a25685 22724105

[80] Borges AB, Abu Hasna A, Matuda AGN, Lopes SR, Mafetano A, Arantes A, et al Adhesive systems effect over bond strength of resin-infiltrated and de-/remineralized enamel. F1000Res. 2019; 8: 1743 10.12688/f1000research.20523.1 31723426 PMC6833993

[81] Attin R, Stawarczyk B, Keçik D, Knösel M, Wiechmann D, Attin T. Shear bond strength of brackets to demineralize enamel after different pretreatment methods. Angle Orthod. 2012; 82(1): 56–61. 10.2319/012311-48.1 21793714 PMC8881030

[82] Perdigão J. Resin infiltration of enamel white spot lesions: An ultramorphological analysis. J Esthet Restor Dent. 2019 10.1111/jerd.12550 31742888

[83] Ehrnford LE, Fransson H. Compressive fracture resistance of the marginal ridge in large Class II tunnels restored with cermet and composite resin. Swed Dent J. 1994; 18(5): 207–211. 7871480

[84] Ji W, Chen Z, Frencken JE. Strength of tunnel-restored teeth with different materials and marginal ridge height. Dent Mater. 2009; 25(11): 1363–1370. 10.1016/j.dental.2009.06.007 19595444

[85] Purk JH, Roberts RS, Elledge DA, Chappell RP, Eick JD. Marginal ridge strength of Class II tunnel restorations. Am J Dent. 1995; 8(2): 75–79. 7546482

[86] de Freitas AR, de Andrada MA, Baratieri LN, Monteiro S Jr, de Sousa CN. Clinical evaluation of composite resin tunnel restorations on primary molars. Quintessence Int. 1994; 25(6): 419–424. 7938431

[87] Hörsted-Bindslev P, Heyde-Petersen B, Simonsen P, Bælum V. Tunnel or saucer-shaped restorations: A survival analysis. Clin Oral Investig. 2005; 9(4): 233–238. 10.1007/s00784-005-0011-6 16133496

[88] Reeh ES, Messer HH, Douglas WH. Reduction in tooth stiffness as a result of endodontic and restorative procedures. J Endod. 1989; 15(11): 512–516. 10.1016/S0099-2399(89)80191-8 2639947

[89] Shahrbaf S, Mirzakouchaki B, Oskoui SS, Kahnamoui MA. The effect of marginal ridge thickness on the fracture resistance of endodontically-treated, composite restored maxillary premolars. Oper Dent. 2007; 32(3): 285–290. 10.2341/06-83 17555181

[90] Strand GV, Tveit AB, Gjerdet NR. Marginal ridge strength of tunnel-prepared teeth restored with various adhesive filling materials. Cem Concr Res. 1999; 29: 645–650. 10.1016/S0008-8846(99)00005-8

[91] Strand GV, Tveit AB, Gjerdet NR, Eide GE. Marginal ridge strength of teeth with tunnel preparations. Int Dent J. 1995; 45(2): 117–123. 7558348

[92] Fasbinder DJ, Davis RD, Burgess JO. Marginal ridge strength in Class II tunnel restorations. Am J Dent. 1991; 4(2): 77–82. 1854444

[93] Roperto R, Sousa YT, Dias T, Machado R, Perreira RD, Leoni GB, et al Biomechanical behavior of maxillary premolars with conservative and traditional endodontic cavities. Quintessence Int. 2019; 50(5): 350–356. 10.3290/j.qi.a42369 30957110

[94] Reeh ES, Douglas WH, Messer HH. Stiffness of endodontically-treated teeth related to restoration technique. J Dent Res. 1989; 68(11): 1540–1544. 10.1177/00220345890680111401 2584522

[95] Strand GV, Nordbø H, Leirskar J, von der Fehr FR, Eide GE. Tunnel restorations placed in routine practice and observed for 24 to 54 months. Quintessence Int. 2000; 31(7): 453–460. 11203966

[96] Ástvaldsdóttir Á, Åhlund K, Holbrook WP, de Verdier B, Tranæus S. Approximal caries detection by DIFOTI: In vitro comparison of diagnostic accuracy/efficacy with film and digital radiography. Int J Dent. 2012; 2012: 326401 10.1155/2012/326401 23213335 PMC3508587

[97] Garrocho-Rangel A, Lozano-Vázquez C, Butrón-Tellez-Girón C, Escobar-García D, Ruíz-Rodriguez S, Pozos-Guillén A. In vitro assessment of retention and microleakage in pit and fissure sealants following enamel pre-etching with sodium hypochlorite deproteinisation. Eur J Paediatr Dent. 2015; 16(3): 212–216. 26418924

[98] Yang F, Mueller J, Kielbassa AM. Surface substance loss of subsurface bovine enamel lesions after different steps of the resinous infiltration technique: A 3D topography analysis. Odontology. 2012; 100(2): 172–180. 10.1007/s10266-011-0031-4 21678019

[99] Lin NJ, Keeler C, Kraigsley AM, Ye J, Lin-Gibson S. Effect of dental monomers and initiators on *Streptococcus mutans* oral biofilms. Dent Mater. 2018; 34(5): 776–785. 10.1016/j.dental.2018.02.003 29523343

[100] Sideridou ID, Karabela MM. Sorption of water, ethanol or ethanol/water solutions by light-cured dental dimethacrylate resins. Dent Mater. 2011; 27(10): 1003–1010. 10.1016/j.dental.2011.06.007 21783239

[101] Finer Y, Santerre JP. Salivary esterase activity and its association with the biodegradation of dental composites. J Dent Res. 2004; 83(1): 22–26. 10.1177/154405910408300105 14691108

[102] Huang B, Siqueira WL, Cvitkovitch DG, Finer Y. Esterase from a cariogenic bacterium hydrolyzes dental resins. Acta Biomater. 2018; 71: 330–338. 10.1016/j.actbio.2018.02.020 29496621 PMC5899954

[103] Nedeljkovic I, De Munck J, Ungureanu AA, Slomka V, Bartic C, Vananroye A, et al Biofilm-induced changes to the composite surface. J Dent. 2017; 63: 36–43. 10.1016/j.jdent.2017.05.015 28554609

[104] Sadeghinejad L, Cvitkovitch DG, Siqueira WL, Santerre JP, Finer Y. Triethylene glycol up-regulates virulence-associated genes and proteins in *Streptococcus mutans*. PLoS One. 2016; 11(11): e0165760 10.1371/journal.pone.0165760 27820867 PMC5098727

[105] Khalichi P, Cvitkovitch DG, Santerre JP. Effect of composite resin biodegradation products on oral streptococcal growth. Biomaterials. 2004; 25(24): 5467–5472. 10.1016/j.biomaterials.2003.12.056 15142727

[106] Nóbrega MTC, Dantas ELA, Alonso RCB, Almeida LFD, Puppin-Rontani RM, de Sousa FB. Hydrolytic degradation of different infiltrant compositions within different histological zones of enamel caries like-lesions. Dent Mater J. 2020 10.4012/dmj.2019-108 31723096

[107] Zantner C, Martus P, Kielbassa AM. Clinical monitoring of the effect of fluorides on long-existing white spot lesions. Acta Odontol Scand. 2006; 64(2): 115–122. 10.1080/00016350500443297 16546854

[108] Moheb A, Misch C, Portnof J, Saveh R, Weiner S. Interproximal invasion as the basis of a Dental Risk Classification: A pilot study. Quintessence Int. 2019; 50(10): 840–847. 10.3290/j.qi.a43234 31538151

[109] Askar H, Schwendicke F, Lausch J, Meyer-Lueckel H, Paris S. Modified resin infiltration of non-, micro- and cavitated proximal caries lesions in vitro. J Dent. 2018; 74: 56–60. 10.1016/j.jdent.2018.03.010 29775637

[110] Tanagawa M, Yoshida K, Matsumoto S, Yamada T, Atsuta M. Inhibitory effect of antibacterial resin composite against *Streptococcus mutans*. Caries Res. 1999; 33(5): 366–371. 10.1159/000016535 10460960

[111] Yoshida K, Tanagawa M, Matsumoto S, Yamada T, Atsuta M. Antibacterial activity of resin composites with silver-containing materials. Eur J Oral Sci. 1999; 107(4): 290–296. 10.1046/j.0909-8836.1999.eos107409.x 10467945

[112] Cheng L, Weir MD, Xu HH, Antonucci JM, Kraigsley AM, Lin NJ, et al Antibacterial amorphous calcium phosphate nanocomposites with a quaternary ammonium dimethacrylate and silver nanoparticles. Dent Mater. 2012; 28(5): 561–572. 10.1016/j.dental.2012.01.005 22305716 PMC3322309

[113] Cheng L, Weir MD, Xu HH, Antonucci JM, Lin NJ, Lin-Gibson S, et al Effect of amorphous calcium phosphate and silver nanocomposites on dental plaque microcosm biofilms. J Biomed Mater Res B Appl Biomater. 2012; 100(5): 1378–1386. 10.1002/jbm.b.32709 22566464 PMC3373271

[114] Bürgers R, Eidt A, Frankenberger R, Rosentritt M, Schweikl H, Handel G, et al The anti-adherence activity and bactericidal effect of microparticulate silver additives in composite resin materials. Arch Oral Biol. 2009; 54(6): 595–601. 10.1016/j.archoralbio.2009.03.004 19375069

[115] Durner J, Stojanovic M, Urcan E, Hickel R, Reichl FX. Influence of silver nano-particles on monomer elution from light-cured composites. Dent Mater. 2011; 27(7): 631–636. 10.1016/j.dental.2011.03.003 21501862

