# Supplementary material for: Ex vivo investigation on internal tunnel approach/internal resin infiltration and external nanosilver-modified resin infiltration of proximal caries exceeding into dentin
Source: PLoS One. 2020 Jan 28;15(1):e0228249. doi: 10.1371/journal.pone.0228249 (PMC6986723; doi:10.1371/journal.pone.0228249)
Supplement: S1 File — (PDF) [file pone.0228249.s001.pdf]

Tooth	PM/M	ICDAS	AgNPs	Pixel	Pixel width	Lesion width	B%	RITC%	FITC%
1	4	3	1	7736660	0,075076	580837,486	0,56	0,32	0,75
7	4	3	1	12310877	0,075076	924251,402	0,88	0,15	0,57
15	4	2	0	5440223	0,075076	408430,182	0,63	0,1	0,27
17	4	3	1	15018636	0,075076	1127539,12	0,63	0,19	0,41
20	4	2	0	9578425	0,075076	719109,835	0,76	0,13	0,53
28	4	3	0	8187091	0,075076	614654,044	0,9	0,31	0,89
32	4	2	1	11262853	0,075076	845569,952	0,68	0,23	0,71
44	4	2	1	16675770	0,075076	1251950,11	0,72	0,15	0,48
51	4	3	0	6343914	0,075076	476275,687	0,73	0,11	0,44
53	4	2	1	11909207	0,075076	894095,625	0,48	0,1	0,27
57	4	3	0	4303365	0,075076	323079,431	0,46	0,12	0,31
68	4	2	0	6668875	0,075076	500672,46	0,6	0,08	0,42
73	4	3	1	8662091	0,075076	650315,144	0,62	0,48	0,86
74	4	2	1	19728164	0,075076	1481111,64	0,53	0,07	0,31
78	4	2	0	7878798	0,075076	591508,639	0,75	0,3	0,55
79	4	2	1	13647958	0,075076	1024634,09	0,53	0,16	0,42
80	4	2	1	3451299	0,075076	259109,724	0,72	0,11	0,59
81	4	3	0	4065309	0,075076	305207,138	0,61	0,12	0,56
105	4	2	1	8767966	0,075076	658263,815	0,57	0,14	0,24
107	4	3	0	8943096	0,075076	671411,875	0,55	0,11	0,43
112	4	3	1	9245715	0,075076	694131,299	0,75	0,16	0,37
115	4	3	0	11990197	0,075076	900176,03	0,86	0,25	0,71
2	5	3	1	6413358	0,075076	481489,265	0,89	0,73	0,86
4	5	2	1	13673470	0,075076	1026549,43	0,85	0,12	0,44
25	5	3	0	12105409	0,075076	908825,686	0,85	0,24	0,59
26	5	3	1	14202652	0,075076	1066278,3	0,71	0,26	0,59
41	5	2	0	3461304	0,075076	259860,859	0,55	0,21	0,39
45	5	3	1	5028372	0,075076	377510,056	0,78	0,24	0,69
46	5	2	1	6607699	0,075076	496079,61	0,91	0,34	0,56
51	5	3	1	10002338	0,075076	750935,528	0,64	0,18	0,47
53	5	2	1	6370040	0,075076	478237,123	0,86	0,22	0,6
55	5	3	1	7827190	0,075076	587634,116	0,97	0,9	0,94
60	5	3	0	11916162	0,075076	894617,778	0,82	0,38	0,58
68	5	2	1	5398623	0,075076	405307,02	0,93	0,35	0,64
88	5	2	0	12467753	0,075076	936029,024	0,68	0,15	0,39
112	5	2	1	9733464	0,075076	730749,543	0,75	0,4	0,61
130	5	3	0	4595813	0,075076	345035,257	0,77	0,16	0,67
134	5	2	1	7958088	0,075076	597461,415	0,74	0,24	0,71
146	5	2	0	7506848	0,075076	563584,12	0,76	0,23	0,6
152	5	3	1	9033278	0,075076	678182,379	0,61	0,07	0,32
155	5	3	0	7748668	0,075076	581738,999	0,79	0,23	0,63
167	5	3	0	11125985	0,075076	835294,45	0,84	0,21	0,82
173	5	2	0	7471064	0,075076	560897,601	0,76	0,14	0,62

Legend

Tooth =	Number of tooth
PM/M =	Molar=4, Premolar=5
ICDAS =	ICDAS 2=2, ICDAS 3=3
AgNPs =	without nanosilver=0, with nanosilver=1
Lesion width	square µm
B% =	Berberine (percentage)
RITC% =	RITC (percentage)
FITC% =	FITC (percentage)
